# New horizons in hearing conditions

**DOI:** 10.1093/ageing/afad150

**Published:** 2023-08-22

**Authors:** Helen Henshaw, Sian Calvert, Eithne Heffernan, Emma E Broome, Clare Burgon, Tom Dening, Kathryn Fackrell

**Affiliations:** National Institute for Health and Care Research (NIHR) Nottingham Biomedical Research Centre, Nottingham NG1 5DU, UK; Hearing Sciences, Mental Health and Clinical Neurosciences, School of Medicine, University of Nottingham, Nottingham NG7 2RD, UK; National Institute for Health and Care Research (NIHR) Nottingham Biomedical Research Centre, Nottingham NG1 5DU, UK; Hearing Sciences, Mental Health and Clinical Neurosciences, School of Medicine, University of Nottingham, Nottingham NG7 2RD, UK; National Institute for Health and Care Research (NIHR) Nottingham Biomedical Research Centre, Nottingham NG1 5DU, UK; Hearing Sciences, Mental Health and Clinical Neurosciences, School of Medicine, University of Nottingham, Nottingham NG7 2RD, UK; National Institute for Health and Care Research (NIHR) Nottingham Biomedical Research Centre, Nottingham NG1 5DU, UK; Hearing Sciences, Mental Health and Clinical Neurosciences, School of Medicine, University of Nottingham, Nottingham NG7 2RD, UK; National Institute for Health and Care Research (NIHR) Nottingham Biomedical Research Centre, Nottingham NG1 5DU, UK; Hearing Sciences, Mental Health and Clinical Neurosciences, School of Medicine, University of Nottingham, Nottingham NG7 2RD, UK; Mental Health and Clinical Neurosciences, School of Medicine, University of Nottingham, Nottingham NG7 2RD, UK; National Institute for Health and Care Research (NIHR) Nottingham Biomedical Research Centre, Nottingham NG1 5DU, UK; Hearing Sciences, Mental Health and Clinical Neurosciences, School of Medicine, University of Nottingham, Nottingham NG7 2RD, UK

**Keywords:** hearing loss, tinnitus, hyperacusis, interprofessional care, older people

## Abstract

Hearing conditions such as hearing loss, tinnitus and hyperacusis are highly prevalent in the population and can severely impact communication and quality of life. Hearing is affected by multiple factors, including heredity, noise exposure, age, sex, ear disorders and lifestyle factors. Globally, hearing loss affects over 80% of adults aged 80 years and older, is often experienced in combination with other long-term health conditions and is a mid-life risk factor for dementia. To form a themed collection, we searched Age and Ageing for articles on hearing conditions published from 2000 onwards. This resulted in 22 articles included within the collection. They examined a range of important topics related to hearing healthcare and research, including noise-induced hearing loss, health service quality and safety, psychological and psychosocial consequences of hearing loss and co-morbidities of hearing loss. All articles reported on hearing loss; there were no published articles with a primary focus on other hearing conditions such as tinnitus or hyperacusis, on the health of older people from the Deaf community or on users of Cochlear implants, suggesting key gaps in knowledge and targets for future research. This New Horizons article highlights novel directions in research and practice and takes a forward look at how research into hearing conditions may develop in years to come. It highlights opportunities for the growth of patient-centred research and hearing healthcare supported by the better integration of health and care services as well as cross-speciality working to include common co-morbid health conditions.

## Key Points

- Hearing conditions are highly prevalent in the population and affect communication and quality of life.
- Hearing loss affects over 80% of adults aged 80 years and older, often in combination with other long-term health conditions.
- For older adults, cochlear implants, the Deaf community, tinnitus and hyperacusis are underrepresented within published research.
- Opportunities for the growth of patient-centred research and care exist, supported by better integration of services.

## Introduction

Hearing is our social sense, on which we rely to connect us with others. However, hearing conditions such as hearing loss, tinnitus and hyperacusis compromise our ability to communicate, and can often co-occur. Hearing conditions are often long-term conditions without cure. They affect a substantial proportion of the global population: with estimates for hearing loss ~20.3% [1], tinnitus ~14.4% [2] and hyperacusis ≤17.2% [3]. Hearing loss refers to reduced hearing sensitivity, ranging from mild to profound [4]. Tinnitus describes the conscious perception of an auditory sensation in the absence of a corresponding external stimulus [5]. Hyperacusis involves a reduced tolerance or increased sensitivity to everyday sounds, whereby they become intense and overwhelming [6].

Hearing conditions increase in both prevalence and severity as people age. For example, tinnitus affects approximately 10% of younger adults, rising to 24% of older adults [2]. Age-related hearing loss (presbycusis), which accounts for the vast majority of hearing loss cases, is acquired over time. It refers to the gradual deterioration of auditory function as a person ages. However, hearing loss is not an inevitable consequence of ageing; there are many modifiable determinants of hearing loss that could reduce its prevalence and severity [7]. Worldwide, over 65% of people aged 60 years or older have hearing loss, rising to >80% of those aged 80 years and older [8, 9]. Deaf individuals (those who identify with Deaf culture and often use sign language to communicate) account for a small percentage of all hearing loss cases. For example, in the UK, there are approximately 12 million people with hearing loss (mild: 26.7%, moderate: 36.8%, severe: 6.3%, profound: 1.3%), with an estimated 24,000 people who use sign language as their main language [8].

Hearing conditions, in particular hearing loss, are associated with a range of negative health conditions including vision loss, cognitive impairment, psychosocial health problems, diabetes, cardiovascular disease, stroke, arthritis and cancer [10] as well as an increased risk of falls [11]. Untreated hearing loss in mid-life has been identified as the leading potentially modifiable risk factor for developing dementia [12]. In 2019, age-related hearing loss was the 3rd largest source of global years lived with disability (YLDs) for adults older than 70 years of age [1].

Estimates from 2019 show that nearly 1 trillion international dollars (Intl$) are lost each year due to unaddressed hearing loss [9]. This includes healthcare costs associated with a failure to address hearing loss (Intl$ 324billion), educational support (Intl$ 27billion), loss of productivity (Intl$ 182.5billion) and societal costs (Intl$ 465.5billion). Furthermore, the communication challenges associated with hearing conditions negatively impact an individual’s ability to manage their health and social care needs. This is particularly true when patients interact with healthcare services [13].

This publication is divided into two parts. First, it describes a ‘themed collection’ of articles published with Age and Ageing that are focused on hearing conditions. Second, it presents a forward-focused discussion of opportunities that exist (a ‘New Horizon’) in research and evidence-based care for older adults with hearing conditions.

To collate the themed collection, we searched Age and Ageing for articles about hearing conditions that were published from 2000 onwards. Our search identified 22 articles eligible for inclusion within the collection. There were no articles identified that had a primary focus on tinnitus, hyperacusis or the Deaf community.

## Looking back: a summary of articles within the themed collection

### Hearing loss and wider health risks

Hearing loss is associated with several long-term health conditions, such as diabetes and vascular disease. What is less clear is the mechanism by which this occurs, which could be due directly to vascular disease or else mediated by various processes, including chronic inflammation (often referred to as inflammageing). Verschuur *et al.* [14] found an association between inflammatory markers and hearing thresholds in over 700 members of the Hertfordshire Ageing Study. This supports the possible importance of inflammation, though over a decade later, the balance of mechanisms is still unresolved.

Another recognised risk factor associated with age-related hearing decline is noise exposure. Hoffs *et al.* [15] found that over a 45-year period (1971–2014) hearing acuity and prevalence of hearing loss among 70-year-old Swedes had improved, for men even more so than women. The improvement in auditory health may reflect the implementation of preventative measures for noise exposure and the decline in industrial-based working environments.

Along with increased co-morbidity, the association between hearing loss and increased mortality is well established, with the risks increasing with more severe hearing loss. For example, Fisher *et al.* [16] found significantly increased all-cause mortality among men with hearing or dual sensory loss, but not with vision loss alone. Cardiovascular disease was the main contributor to excess deaths. Liljas *et al.* [17] also examined the incidence of activities of daily living (ADL) disability as well as mortality among older men, reporting increased risks of incident ADL difficulties, especially in men who still had hearing problems despite using hearing aids. They also found that the association with increased mortality was no longer significant after adjusting for covariates, but as these covariates included mobility and ADL problems, perhaps this is unsurprising.

### Health service quality and safety for adults with hearing loss

People living in the community with hearing loss may require additional support to manage their overall health. A longitudinal study by Schneider *et al.* [18] investigated the impact of hearing loss on formal community support services and informal caregivers. Hearing loss was associated with greater reliance on both types of support. Individuals who did not use their hearing aids were twice as likely to require community support compared with those without hearing loss.

Older adults living with sensory impairment may face difficulties managing their medication stemming from barriers to communication. In a community-based study, Smith *et al.* [19] reported challenges for individuals with hearing loss, including difficulty ordering medication over the telephone and misunderstanding medication information received from pharmacists. Therefore, this patient group is at greater risk of experiencing medication errors and/or adverse events.

The quality of care that older people with hearing loss experience in social care settings is also negatively impacted by communication difficulties. An ethnographic study of two residential care homes in the UK found that social and environmental factors influenced residents’ access to social opportunities [20]. Social interactions between residents and care staff were predominately task-focused and limited in choice. Background noise, such as music or television, hampered residents’ ability to communicate with others.

Furthermore, healthcare professionals may be ill-equipped to address the communication challenges posed by hearing loss, thus influencing the quality of care that patients receive. Smith *et al.* [21] explored healthcare professionals’ experiences communicating with adults with hearing loss through an online survey. Healthcare professionals from both primary and specialist care services reported that hearing loss negatively impacted the perceived quality of care their patients received. They also noted a lack of formal training opportunities for communicating with this patient group, particularly among primary care clinicians, and insufficient familiarity with resources to support patients’ communication needs.

### Psychological and psychosocial consequences of hearing loss

The psychological and psychosocial impact of hearing loss is profound, persistent and increases with hearing loss severity. Gopinath *et al.* [22] examined ADL 1,572 adults aged ≥60 years, including those with (*n* = 686) and without (*n* = 886) hearing loss, from the 1997–2004 Australian Blue Mountains Hearing Study. A significantly higher proportion of adults with hearing loss, compared with those without, experienced difficulties with three out of seven basic activities (walking, bathing and continence) and six out of seven instrumental activities (using the telephone, shopping, travelling, preparing meals, housework and taking medicine). Additionally, greater hearing loss severity was associated with impaired ADL, following adjustments for key factors (e.g. age, cognitive impairment, depressive symptoms).

Gopinath *et al.* [23] also used Blue Mountains Hearing Study data to examine the temporal relationship between hearing loss and self-reported, hearing-related emotional (e.g. frustration, embarrassment) and social difficulties (e.g. listening problems, limited social life). The sample comprised 811 adults aged ≥55 years who were assessed on two occasions (1997–99 and 2002–04). Approximately, two out of three participants with hearing loss at baseline developed significant hearing-related emotional and social difficulties within 5 years. These difficulties were associated with higher odds of poor self-rated health, depressive symptoms and poor quality of life following adjustments for key factors. These studies by Gopinath and colleagues suggest that effective aural rehabilitation is needed to maintain functional independence and psychosocial wellbeing and to reduce reliance on support services in later life.

Cosh *et al.* [24] explored the relationships between hearing loss, anxiety symptoms and generalised anxiety disorder (GAD) in adults aged 65 and above (*n* = 3,928) from the Three-City study in France. Baseline hearing loss was associated with self-reported anxiety symptoms but not incident GAD. In contrast, GAD at baseline was associated with an increased risk of self-reported hearing loss at follow-up. Therefore, perhaps managing anxiety may reduce the psychosocial impact of hearing loss.

Dobrota *et al.* [25] studied self-reported hearing problems, social networks and depressive symptoms in a sample of almost 6,000 over 9 years. Interestingly, prevalent hearing problems were associated with depression, whereas incident hearing problems led to a significant decline in the strengths of participants’ social networks, especially in women. This seems to reflect well how newly developing hearing problems are likely to affect individuals.

Finally, Heffernan *et al.* [26] used a qualitative approach to explore the perspectives of adults living with hearing loss and audiologists in the UK regarding hearing-related social isolation. Their findings suggest hearing loss can negatively affect social engagement, roles, identity and relationships, potentially leading to further negative consequences (e.g. stigmatisation, depression). Few interventions specifically target hearing-related social isolation, and audiologists may lack the resources, training and time needed to deliver them. Ideally, a hearing-related social isolation intervention would be patient-centred, patient-led, delivered in the community and accessible to all (e.g. people with mobility difficulties or limited computer literacy).

### Dual sensory impairment

Dual sensory impairment (e.g. experiencing both vision and hearing loss) is common in older adults, and with an ageing population the prevalence is expected to increase [27]. Dual sensory impairment is associated with poor health outcomes, including poor mental health, cognitive decline and increased mortality [16, 28, 29]. Fisher *et al.* [16] examined the relationship between hearing and vision impairments and mortality in a large population cohort study (*n* = 4,926) and found that older adults with dual-sensory impairments had higher rates of depression, cognitive impairments and diabetes compared with those without impairments. Overall, dual sensory impairments were associated with an increased risk of all-cause mortality compared with those with hearing or vision loss alone. Using data collected from three international surveys of ageing (*n* = 45,805), Maharani *et al.* [28] found that sensory impairment in older adults consistently showed a trajectory of cognitive decline, those with dual sensory impairments declining faster, performing less well on the cognitive tests and being less socially engaged than those with no or single impairment. Parada *et al.* [29] examined associations between vision and hearing loss and age-related cognitive decline within a longitudinal study of 1,383 adults who were followed for up to 25 years (mean = 7.3 years). Both vision and hearing loss were shown to be associated with a more rapid decline in cognitive function with ageing.

Due to the complexity of dual sensory impairments, more comprehensive environmental adaptations, management and assessments are required than for vision or hearing loss alone. In a study exploring healthcare professionals’ perceptions of the assessment and service provision for dual sensory impairments and cognition, Leroi *et al.* [30] identified the lack of adapted assessments for concurrent deficits and limited interdisciplinary support and communication between professionals as key service elements that need improvement. While professionals in dementia and/or hearing services commonly asked about other impairments, vision professionals were less likely to ask. The authors concluded that cross-discipline training and guidelines are needed to improve assessment and care pathways. Furthermore, in relation to pharmaceutical care for older adults with dual sensory impairments, Smith *et al.* [19] identified potential barriers in ordering, storing and administering medicines with this population and recommended a system-wide approach to ensure that continuous, safe integrated pharmaceutical care is provided for all.

### Hearing loss, cognitive decline and dementia

Untreated hearing loss in mid-life is a potentially modifiable risk factor for dementia, accounting for approximately 8% of dementia cases globally [12]. Proposed mechanisms linking the two include: hearing-loss induced impoverished environment, increased cognitive demand, common pathology or an interaction between pathology and increased cognitive demand [31]. A 5th mechanism (the auditory brain) suggests that healthy ageing affects multiple stages of auditory processing, and that compensatory mechanisms in peripheral and central (executive control) systems may compensate until compromised by neurodegenerative pathology [32]. Hearing loss is ‘frequently unidentified and is a clear modifiable risk factor to promote brain health’ [p.iii17, 33]. Using data from five waves of the English Longitudinal Study of England and the Health and Retirement Study, Matthews *et al.* [34] found that consistently reporting good hearing was associated with better cognitive function (episodic memory). These results remained significant after controlling for several potentially confounding variables (age, sex, marital status, education, wealth, smoking and drinking status, physical activity and self-reported general health). Furthermore, two studies included within the previous section [28, 29] reported more rapid rates of cognitive decline in people with dual sensory impairment, compared with vision or hearing loss alone.

For people living with dementia, poor hearing is known to exacerbate the effects of cognitive decline. Naylor *et al.* [35] analysed health records data from 380,794 veterans who obtained hearing aids from the Veterans Affairs healthcare system in the United States. Analyses showed a protective effect of hearing aids against dementia, but also that better cognitive function predisposed persistent hearing aid use. Thus, research studies targeting a protective effect of hearing aids for dementia must consider possible factors underlying hearing aid non-use. In a 2003 study of patients with primary dementia [36], 31 participants were fitted with hearing aids, which were well accepted (used every day or most days), of which, 42% showed an improvement on an independently rated measure of change, and both carers and participants reported an overall reduction in activity limitations arising from hearing loss. The authors concluded that the presence of dementia should not preclude assessment for hearing aids.

An important consideration is that screening tools designed to identify cognitive impairment frequently rely on auditory-based assessment methods, raising concerns about the over-diagnosis of dementia in people with hearing loss [37]. DeSilva *et al.* [38] found no difference in performance on a written compared with the standard Mini-Mental Status Examination (a popular cognitive screening tool) in people with moderate to severe hearing loss, though the written version was preferred. A survey by Omar *et al.* [39] explored current practices and views among UK professionals regarding hearing assessment and care in memory clinics, as well as cognitive assessment and care in hearing aid clinics. Results showed that although professionals working in memory and audiology services felt addressing this co-morbidity would be useful, current practice varies and does not generally address it.

## Looking forward: a new horizon in hearing conditions

The number of people living with hearing conditions is increasing, placing a greater burden on health and social care services and impacting the quality of care that individuals receive [13]. Older people living with hearing conditions experience increased rates of co-morbidity, making them more frequent healthcare users [40]. To improve the quality of care for people living with hearing loss, tinnitus and hyperacusis, communication challenges need to be addressed to ensure that this group of patients are cared for in an appropriate and safe manner across all health and social care services [e.g. 41].

Government policies, such as the UK Accessible Information Standard provide a legal requirement to ensure that the needs of people who have hearing loss, are Deaf or blind, or experience dual sensory loss are met when using health and social care services [42]. In 2015, the UK Department of Health and Social Care Action Plan on Hearing Loss [43] called for the timely identification, diagnosis and management of hearing conditions to reduce negative outcomes for patients and health and care services. Comprehensive guidance is offered via the National Institute for Health and Care Excellence (NICE) [44, 45] and the Royal College of General Practitioner’s [46, 47]. Furthermore, an innovative service delivery model highlights the benefits of integrating audiology triage into primary care [48]. Finally, the World Health Organization ‘World Report on Hearing’ [9] was developed to aid integration of ear and hearing care into its’ Member States national health plans.

Hearing loss is the 3rd leading cause of YLD, and the leading cause of YLD for those over 70 years of age [1]. Yet, funding invested into hearing research is disproportionally low compared with the disease burden [49]. The research community should strive to address unanswered priority research questions, e.g. those arising from NICE guidance for the assessment and management of hearing loss [44] and tinnitus [45], to tackle key gaps in evidence required to guide evidence-based patient care. Research priority setting exercises with diverse user involvement (e.g. patients, carer and clinicians) are important in identifying where research would make the most difference for those with lived and daily experience of health conditions. For example, the James Lind Alliance is a non-profit making initiative that enables health research funders and commissioners to be aware of the issues that most matter to the people who need to use the research findings in their everyday lives [50]. Priorities for research have been established via James Lind Alliance Priority Setting Partnerships for mild–moderate hearing loss [51], tinnitus [52], hyperacusis [6] and aspects of balance (which has not been considered directly within this themed collection) [53]. Furthermore, international of Core Outcome Sets for tinnitus [54] and single sided deafness [55] define important outcomes to measure in clinical trials, as identified and agreed upon by key international stakeholders (patients, healthcare professionals, researchers and industry representatives).

Moving forward, hearing research is on a trajectory from a single- to multiple-condition foci. This will enable health and care professionals to deliver future evidence-based care informed by the experiences of those who live with (often co-morbid) hearing conditions alongside other (potentially multiple) long-term health conditions. Hearing healthcare professionals recognise the potential benefits of addressing common co-morbidities of hearing (such as dementia) via integrated care pathways [39, 56]. An example of innovation in this area is the exploration of integrating audiology and memory services [57, 58]. Finally, a new James Lind Alliance Priority Setting Partnership in co-existing dementia and hearing conditions is the first to bring together two specific disease areas. The partnership will involve patients, carers and clinicians in identifying priority uncertainties about the prevention, diagnosis and treatment of these co-existing long-term ‘umbrella’ conditions [59]. The partnership also emphasises the representation of individuals and groups who may be typically underserved in research (e.g. unpaid carers, individuals from ethnic minority groups, people with lived experience of hearing loss and dementia and those living in residential care settings).

## Concluding remarks

This publication summarises a themed collection on hearing conditions that offers a broad overview of key topics related to the consequences, diagnosis, management and common co-morbidities of hearing loss. There was a complete absence of articles reporting on older members of the Deaf community. Articles on the use of cochlear implants and on hearing conditions other than hearing loss are lacking within the journal’s themed collection, highlighting gaps in knowledge and key targets for future research.

Clinicians should be aware of the high prevalence of hearing conditions in older adults to aid the effective diagnosis, assessment and management of hearing conditions as well as assess risks for, and management of, associated chronic co-morbid conditions. A new horizon of opportunities for the growth of patient-centred evidence-based care exists, supported by targeted priority research and the integration of health and care services, using an interprofessional approach.

Finally, what might we include when writing a similar article in 10 years’ time? We will know more about the role of hearing rehabilitation in dementia prevention, patients will have greater choice in hearing intervention and self-management support, the use of cochlear implants will be more widespread, audiology and other health and care services will have worked out how to work best together and basic science will have delivered some major advances, for example the regeneration of cochlear hair cells.

## Supplementary Material

Hearing_conditions_collection_afad150
