# Supplementary material for: New horizons in hearing conditions
Source: Age Ageing. 2023 Aug 22;52(8):afad150. doi: 10.1093/ageing/afad150 (PMC10442518; doi:10.1093/ageing/afad150)
Supplement: Hearing_conditions_collection_afad150 [file hearing_conditions_collection_afad150.docx]

Hearing conditions

Hearing conditions such as hearing loss, tinnitus and hyperacusis are highly prevalent in the population and can severely impact communication and quality of life. Hearing is affected by multiple factors, including heredity, noise exposure, age, sex, ear disorders and lifestyle factors. Globally, hearing loss affects over 80% of adults aged 80 years and older, is often experienced in combination with other long-term health conditions, and is a mid-life risk factor for dementia.

To form the themed collection, we searched *Age and Ageing* for articles on hearing conditions published from 2000 onwards. This resulted in 22 articles included within the collection. They examined a range of important topics related to hearing healthcare and research, including noise-induced hearing loss, health service quality and safety, psychological and psychosocial consequences of hearing loss, and comorbidities of hearing loss. All articles reported on hearing loss; there were no published articles with a primary focus on other hearing conditions such as tinnitus or hyperacusis, on the health of older people from the Deaf community, or on users of Cochlear implants, suggesting key gaps in knowledge and targets for future research.

A New Horizons article, [New Horizons in Hearing conditions](https://academic.oup.com/ageing/article/52/8/afad150/7246474), was commissioned to accompany this *Age and Ageing* themed collection, which you can read in full for free.

**Helen Henshaw, Sian Calvert, Eithne Heffernan, Emma E. Broome, Clare Burgon, Tom Dening, Kathryn Fackrell.**

The collection

1. Allen NH, Burns A, Newton V, Hickson F, Ramsden R, Rogers J, et al. The effects of improving hearing in dementia. Age Ageing. Volume 32, Issue 2, March 2003, Pages 189–193, <https://doi.org/10.1093/ageing/32.2.189>
2. Cosh, S., Naël, V., Carrière, I., Daien, V., Amieva, H., Delcourt, C., ... & Sense-Cog Consortium. (2018). Bidirectional associations of vision and hearing loss with anxiety: prospective findings from the Three-City Study. Age and Ageing, 47(4), 582-589. <https://doi.org/10.1093/ageing/afy062>
3. De Silva, M. L., Mclaughlin, M. T., Rodrigues, E. J., Broadbent, J. C., Gray, A. R., & Hammond-Tooke, G. D. (2008). A Mini-Mental Status Examination for the hearing impaired. Age and ageing, 37(5), 593-595. <https://doi.org/10.1093/ageing/afn146>
4. Dobrota, S. D., Biggs, M. L., Pratt, S., Popat, R., & Odden, M. C. (2022). The association of hearing problems with social network strength and depressive symptoms: the cardiovascular health study. Age and Ageing, 51(8), afac181. <https://doi.org/10.1093/ageing/afac181>
5. Fisher, D., Li, C. M., Chiu, M. S., Themann, C. L., Petersen, H., Jónasson, F., ... & Cotch, M. F. (2014). Impairments in hearing and vision impact on mortality in older people: the AGES-Reykjavik Study. Age and ageing, 43(1), 69-76. <https://doi.org/10.1093/ageing/aft122>
6. Gopinath, B., Schneider, J., McMahon, C. M., Teber, E., Leeder, S. R., & Mitchell, P. (2012). Severity of age-related hearing loss is associated with impaired activities of daily living. Age and Ageing, 41(2), 195-200. <https://doi.org/10.1093/ageing/afr155>
7. Gopinath, B., Hickson, L., Schneider, J., McMahon, C. M., Burlutsky, G., Leeder, S. R., & Mitchell, P. (2012). Hearing-impaired adults are at increased risk of experiencing emotional distress and social engagement restrictions five years later. Age and Ageing, 41(5), 618-623. https://doi.org/10.1093/ageing/afs058
8. Heffernan, E., Withanachchi, C. M., & Ferguson, M. A. (2022). ‘The worse my hearing got, the less sociable I got’: a qualitative study of patient and professional views of the management of social isolation and hearing loss. Age and Ageing, 51(2), afac019. <https://doi.org/10.1093/ageing/afac019>
9. Hoff, M., Tengstrand, T., Sadeghi, A., Skoog, I., & Rosenhall, U. (2018). Improved hearing in Swedish 70-year olds—a cohort comparison over more than four decades (1971–2014). Age and ageing, 47(3), 437-444. <https://doi.org/10.1093/ageing/afy002>
10. Leroi, I., Himmelsbach, I., Wolski, L., Littlejohn, J., Jury, F., Parker, A., ... & (SENSE-Cog Expert Reference Group). (2019). Assessing and managing concurrent hearing, vision and cognitive impairments in older people: an international perspective from healthcare professionals. Age and ageing, 48(4), 580-587. <https://doi.org/10.1093/ageing/afy183>
11. Liljas, A. E., Wannamethee, S. G., Whincup, P. H., Papacosta, O., Walters, K., Iliffe, S., ... & Ramsay, S. E. (2016). Hearing impairment and incident disability and all-cause mortality in older British community-dwelling men. Age and Ageing, 45(5), 662-667. <https://doi.org/10.1093/ageing/afw080>
12. Maharani, A., Dawes, P., Nazroo, J., Tampubolon, G., Pendleton, N., & Sense-Cog WP1 group. (2018). Visual and hearing impairments are associated with cognitive decline in older people. Age and ageing, 47(4), 575-581. <https://doi.org/10.1093/ageing/afy061>
13. Matthews, K., Dawes, P., Elliot, R., Pendleton, N., Tampubolon, G., & Maharani, A. (2023). Trajectories of self-reported hearing and their associations with cognition: evidence from the United Kingdom and United States of America. Age and ageing, 52(2), afad017. <https://doi.org/10.1093/ageing/afad017>
14. Naylor, G., Dillard, L., Orrell, M., Stephan, B. C., Zobay, O., & Saunders, G. H. (2022). Dementia and hearing-aid use: a two-way street. Age and Ageing, 51(12), afac266. <https://doi.org/10.1093/ageing/afac266>
15. Omar R, Kuo L, Costafreda SG, Hall A, Forbes M, O’Brien JT, et al. Managing comorbid cognitive impairment and hearing loss in older adults: a UK survey of audiology and memory services. Age Ageing, Volume 52, Issue 5, May 2023, 52(5), afad080. <https://doi.org/10.1093/ageing/afad080>
16. Parada, H., Laughlin, G. A., Yang, M., Nedjat-Haiem, F. R., & McEvoy, L. K. (2021). Dual impairments in visual and hearing acuity and age-related cognitive decline in older adults from the Rancho Bernardo Study of Healthy Aging. Age and Ageing, 50(4), 1268-1276. <https://doi.org/10.1093/ageing/afaa285>
17. Pryce, H., & Gooberman-Hill, R. (2012). ‘There's a hell of a noise’: living with a hearing loss in residential care. Age and Ageing, 41(1), 40-46. <https://doi.org/10.1093/ageing/afr112>
18. Schneider, J., Gopinath, B., Karpa, M. J., McMahon, C. M., Rochtchina, E., Leeder, S. R., & Mitchell, P. (2010). Hearing loss impacts on the use of community and informal supports. Age and ageing, 39(4), 458-464. <https://doi.org/10.1093/ageing/afq051>
19. Smith, A., Macaden, L., Kroll, T., Alhusein, N., Taylor, A., Killick, K., ... & Watson, M. (2019). A qualitative exploration of the experiences of community dwelling older adults with sensory impairment/s receiving polypharmacy on their pharmaceutical care journey. Age and ageing, 48(6), 895-902. <https://doi.org/10.1093/ageing/afz092>
20. Smith, S., Manan, N. S. I. A., Toner, S., Al Refaie, A., Müller, N., Henn, P., & O’Tuathaigh, C. M. (2020). Age-related hearing loss and provider-patient communication across primary and secondary care settings: a cross-sectional study. Age and Ageing, 49(5), 873-877. <https://doi.org/10.1093/ageing/afaa041>
21. Vassilaki, M., Aakre, J. A., Knopman, D. S., Kremers, W. K., Mielke, M. M., Geda, Y. E., ... & Petersen, R. C. (2019). Informant-based hearing difficulties and the risk for mild cognitive impairment and dementia. Age and Ageing, 48(6), 888-894. <https://doi.org/10.1093/ageing/afz099>
22. Verschuur, C. A., Dowell, A., Syddall, H. E., Ntani, G., Simmonds, S. J., Baylis, D., ... & Aihie Sayer, A. (2012). Markers of inflammatory status are associated with hearing threshold in older people: findings from the Hertfordshire Ageing Study. Age and Ageing, 41(1), 92-97. <https://doi.org/10.1093/ageing/afr140>
